# Statement of Retraction: Circular RNA CNOT2 knockdown regulates twist family BHLH transcription factor via targeting microRNA 409-3p to prevent breast cancer invasion, migration and epithelial–mesenchymal transition

**DOI:** 10.1080/21655979.2024.2401722

**Published:** 2024-09-12

We, the Publisher of the journal *Bioengineered*, have retracted the following article:

Su, Q., Shen, H., Gu, B., & Zhu, N. (2021). Circular RNA CNOT2 knockdown regulates twist family BHLH transcription factor via targeting microRNA 409-3p to prevent breast cancer invasion, migration and epithelial–mesenchymal transition. *Bioengineered, 12*(1), 9058–9069. https://doi.org/10.1080/21655979.2021.1974805

Since publication, significant concerns have been raised about the integrity of the data, the reported methodology and reported results in the article.

When approached for an explanation, the authors were unable to provide an explanation, their original data or the protocols used.

As verifying the validity of published work is core to the integrity of the scholarly record, we are therefore retracting the article. All authors listed in this publication have been informed.

We have been informed in our decision-making by our editorial policies and the COPE guidelines.

The retracted article will remain online to maintain the scholarly record, but it will be digitally watermarked on each page as ‘Retracted’.

